# A Comprehensive RNA Study to Identify circRNA and miRNA Biomarkers for Docetaxel Resistance in Breast Cancer

**DOI:** 10.3389/fonc.2021.669270

**Published:** 2021-05-14

**Authors:** Peide Huang, Fengyu Li, Zongchao Mo, Chunyu Geng, Fang Wen, Chunyan Zhang, Jia Guo, Song Wu, Lin Li, Nils Brünner, Jan Stenvang

**Affiliations:** ^1^ BGI, BGI-Shenzhen, Shenzhen, China; ^2^ BGI Genomics, BGI-Shenzhen, Shenzhen, China; ^3^ MGI, BGI-Shenzhen, Shenzhen, China; ^4^ Shenzhen Luohu Hospital Group, The Affiliated Luohu Hospital of Shenzhen University, Shenzhen, China; ^5^ National Research Center for Translational Medicine, National Key Scientific Infrastructure for Translational Medicine, Shanghai Jiao Tong University, Shanghai, China; ^6^ Department of Drug Design and Pharmacology, Faculty of Health and Medical Sciences, University of Copenhagen, Copenhagen, Denmark

**Keywords:** breast cancer, docetaxel resistance, RNA sequencing, circRNA, miRNA

## Abstract

To investigate the relationship between non-coding RNAs [especially circular RNAs (circRNAs)] and docetaxel resistance in breast cancer, and to find potential predictive biomarkers for taxane-containing therapies, we have performed transcriptome and microRNA (miRNA) sequencing for two established docetaxel-resistant breast cancer (DRBC) cell lines and their docetaxel-sensitive parental cell lines. Our analyses revealed differences between circRNA signatures in the docetaxel-resistant and -sensitive breast cancer cells, and discovered circRNAs generated by multidrug-resistance genes in taxane-resistant cancer cells. In DRBC cells, circABCB1 was identified and validated as a circRNA that is strongly up-regulated, whereas circEPHA3.1 and circEPHA3.2 are strongly down-regulated. Furthermore, we investigated the potential functions of these circRNAs by bioinformatics analysis, and miRNA analysis was performed to uncover potential interactions between circRNAs and miRNAs. Our data showed that circABCB1, circEPHA3.1 and circEPHA3.2 may sponge up eight significantly differentially expressed miRNAs that are associated with chemotherapy and contribute to docetaxel resistance *via* the PI3K-Akt and AGE-RAGE signaling pathways. We also integrated differential expression data of mRNA, long non-coding RNA, circRNA, and miRNA to gain a global profile of multi-level RNA changes in DRBC cells, and compared them with changes in DNA copy numbers in the same cell lines. We found that Chromosome 7 q21.12-q21.2 was a common region dominated by multi-level RNA overexpression and DNA amplification, indicating that overexpression of the RNA molecules transcribed from this region may result from DNA amplification during stepwise exposure to docetaxel. These findings may help to further our understanding of the mechanisms underlying docetaxel resistance in breast cancer.

## Introduction

Breast cancer is the most prevalent and mortal cancer among women worldwide (1). Chemotherapy is a very important treatment for breast cancer, especially for hormone-insensitive, advanced or metastatic breast cancer.

The taxane class of chemotherapy agents, including docetaxel and paclitaxel, were introduced into the treatment of advanced breast cancer about two decades ago and recognized as a significant improvement for breast cancer treatment (2). Paclitaxel is a derivative of the crude extract of the pacific yew tree *(Taxus brevifolia)* (3). Docetaxel is a semisynthetic derivative of paclitaxel, and regarded as a second-generation taxane (4).

The anti-cancer mechanism for taxanes involves binding and stabilizing GDP-bound tubulin (5, 6), and thus causing cell cycle arrest and apoptosis of cancer cells (7). Taxane-containing treatment regimens have been shown to improve overall survival in both early-stage and advanced breast cancer when compared with non-taxane regimens (8, 9). Taxanes, either alone or in combination, are commonly applied as first-line treatments for advanced breast cancer (10). However, many patients receiving taxanes develop drug resistance, which might be a result of mutations or dysregulation of transcription in drug-resistance genes, or post-transcriptional mechanisms (11–14). However, no biomarkers have been successfully identified for response to taxanes in clinical treatments of breast cancer.

Circular RNAs (circRNAs) are covalently closed RNA molecules, usually generated from canonical splice sites and comprised of exonic sequences (15). Their unique structure allows them to escape from exonuclease-mediated degradation and they therefore become more stable than linear RNAs (16). Due to the lack of free ends, circRNAs are not capped and thus are not predicted to be translated by cap-dependent mechanisms. Thus, they are classified as non-coding RNAs (17). Although the functions of circRNAs remain largely unknown, recent studies have revealed that circRNAs may participate in biological processes that include affecting mRNA expression by competing with linear splicing (18), binding and sequestering certain proteins (19), and functioning as microRNA sponges (20). So far, the most well-documented function of circRNAs is their ability to act as molecular sponges to bind microRNAs (miRNAs) and thus reduce inhibition of their target genes. For example, ciRS-7 (circular RNA sponge for miR-7) was found to contain over 70 miRNA binding sites and to strongly reduce miR-7 activity (20). Among 9 miRNAs bound by circHIPK3, inhibition of miR-124 activity has been shown to promote human cell growth (21). Also, evidence is accumulating that circRNAs are involved in the development of cancer and other diseases. For example, circTCF25 has been shown to sponge up miR-107 and miR-103a-3p, thereby increasing expression of CDK6, and to promote migration and proliferation of bladder cancer cells (22). Differential expression of circRNAs is also involved in radioresistance of esophageal cancer (23) and chemotherapy resistance of acute myeloid leukemia and breast cancer (24, 25). These findings point to circRNAs as potential biomarkers for diagnosis and resistance to treatment of cancer.

To investigate possible relationships between circRNAs and docetaxel resistance in breast cancer, we performed RNA sequencing and circRNA analysis of two cell lines, MDA-MB-231 and MCF-7, and their docetaxel-resistant cell sublines MDA-RES and MCF7-RES. We also performed miRNA sequencing to explore potential interactions between circRNAs and miRNAs. Our data show that circRNAs may sponge up chemotherapy-associated miRNAs and regulate signaling pathways that contribute to docetaxel resistance in breast cancer cells.

## Materials and Methods

### Cell Culture and Treatments

Two docetaxel-resistant human breast cancer cell lines, MDA-RES and MCF7-RES, and their parental cells, MDA-MB-231 and MCF-7, were obtained from Hansen et al. (12). Cells were cultured in DMEM (Gibco, ThermoFisher Scientific, Massachusetts, USA) containing L-glutamine, supplemented with 10% fetal calf serum (FCS) (Gibco, ThermoFisher Scientific, Massachusetts, USA) for MDA-MB-231, or 5% FCS and 1% non-essential amino acids (Gibco, ThermoFisher Scientific, Massachusetts, USA) for MCF-7. Cells were kept in a humidified atmosphere containing 5% carbon dioxide at 37°C. MCF-RES and MDA-RES cells were maintained in growth medium with 65 and 150 nM docetaxel, respectively.

### Total RNA Purification and rRNA-Depleted Transcriptome Sequencing

Total RNA from 3 passages (3 biological replicates) each of MCF-7, MCF7-RES, MDA-MB-231 and MDA-RES cells was purified using the RNAiso™ Plus Kit (TaKaRa, Japan). After RNA purification and DNase I digestion, ribosomal RNAs (rRNAs) were removed from total RNA with the RiboMinus Eukaryote Kit (Qiagen, Valencia, CA). The remaining RNAs were fragmented and used to synthesize cDNAs, followed by end repairing and adenine connection. Then the sequencing adaptors were ligated to the fragments and those with suitable sizes were selected for PCR amplification. An ABI StepOnePlus System (ThermoFisher Scientific, Massachusetts, USA) and an Agilent 2100 Bioanaylzer (Agilent Technologies, California, USA) were used to quantify and qualify the sample libraries in the quality control steps. Finally, all the libraries were sequenced by an Illumina HiSeqTM 2000 sequencer.

### Detection and Quantification of circRNAs

The raw sequencing reads were cleaned by cutting adaptor sequences and filtering low quality reads that contained more than 50% low quality bases (base quality < 10) and 5% undefined nucleotides [NT]. To avoid introducing error into the next analysis by reads mapped to the remaining rRNAs, we aligned reads to vertebrate rRNA sequences and filtered the perfect mapping reads. After this two-step filtering, the remaining reads were considered clean and used in the subsequent expression profile. Since circRNA is produced by the junction of the downstream spliced donor and upstream spliced acceptor sites, called “back-spliced sites”, we used Tophat2 software (26) to distinguish the back-spliced reads from the clean reads. CIRCexplorer software (27) uses back-spliced reads and NCBI RefSeq gene annotations as input to identify circRNA. We kept only exonic circRNAs for the next analysis. To quantify the relative expression of circRNAs, the total number of reads mapped to hg19 were calculated for each sample, and then the number of back-spliced reads for each circRNA was normalized by the total number of mapped reads and the read length. The relative expression level for each circRNA was denoted as spliced reads per billion mappings (SRPBM) (21).

### Validation of circRNAs

Outward-facing primers were designed for circRNAs by using Primer 5.0. The PCR products across the junction points ranged from 200 bp to 500 bp. GAPDH served as an endogenous control. Total RNA from each cell line was extracted using RNAiso Plus (TAKARA, Japan). qPCR was performed in triplicate (n=3) on a StepOnePlus instrument (Applied Biosystems, Thermo Fisher Scientific, USA) using the SYBR Premix Ex Taq II reagent (TAKARA, Japan). The relative expression of circRNAs were calculated with the 2^−ΔΔCt^ approach. Finally, the sequences of the PCR products were detected by a 3730xl DNA Analyzer (ThermoFisher Scientific, Massachusetts, USA) and then mapped to reference genome hg19 to validate the exact junction points and the cyclization events.

### miRNA Binding-Site Prediction

Using circRNA annotation information provided by CIRCexplorer software, we extracted the sequences of circABCB1, circEPHA3.1, and circEPHA3.2 from the human reference genome hg19 using bedtools software (28). Then, we used 4 software tools, TargetScan v7.1 (29), Miranda (30), Pita (31), and RNAhybrid (32) to predict potential microRNA target sites on the 3 circRNAs. The sequences of the miRNAs were downloaded from miRBase database v20 (http://www.mirbase.org/ftp.shtml), which contains all mature human microRNA sequences (2794 entries in total).

### miRNA Extraction and Sequencing

The total RNA samples from 3 passages (3 biological replicates) of the MCF-7, MCF7-RES, MDA-MB-231 and MDA-RES cell lines were purified using the RNAiso™ Plus Kit (TaKaRa, Japan). 1μg total RNA from each sample was subjected to PAGE gel separation, and the stripes of 18-30 NT were selected and recycled. The small RNA libraries were constructed using the MGIEasyTM Small RNA Library Prep Kit V1 (MGI, Cat No. 85-05535-00). Briefly, the 3’ and 5’ adaptors were ligated to the recycled RNA. Then, the cDNAs were prepared and PCR amplification was performed for 16 cycles. PCR products were purified on 6% acrylamide gels followed by elution and ethanol precipitation, and then quantified using a Qubit fluorometer (ThermoFisher Scientific, Massachusetts, USA) and pooled together to produce single-strand DNA (ssDNA) circles. Then the ssDNA circles were used to produce DNA nanoballs (DNBs) *via* rolling circle replication to amplify the fluorescent signals during the sequencing process. Then the DNBs were loaded into sequencing chips, and 50 bp single-end reads were generated on a BGISEQ-500 platform (BGI, Shenzhen, China) for subsequent analysis.

### miRNA Sequencing Data Analysis

Clean reads were aligned to the mature miRNA sequences downloaded from the miRBase database v20 (http://www.mirbase.org/) and BLAST (basic local alignment search tool) was used to identify known miRNAs. To quantify miRNA expression levels, we counted read numbers mapped to each miRNA. Thus, we obtained an miRNA expression matrix. To find miRNAs differentially expressed between groups, we used the DESeq2 (33) software package to analyze the expression matrix in the R environment. The P value was calculated based on a negative binomial distribution model and adjusted by the Benjamini-Hochberg method (33).

### Chemotherapy-Associated miRNA Searching and Information Formatting

The search terms “chemotherapy” and “miRNA” were used for a literature search in the PubMed databases. The abstracts of the literature identified in the search were download and formatted with a custom Perl script to form a reference list for chemotherapy-related miRNA. We used the Pharmaco-miR database (http://www.pharmaco-mir.org/), which links miRNAs with drug effects, to download a list of miRNAs related to the drugs docetaxel and paclitaxel. Finally, we combined the PubMed references and Pharmaco-miR miRNA list to form an annotation data source (named Reported.miR, **Supplementary Table 6**) for further investigation of the relationships between miRNAs and chemotherapy.

### miRNA Target Gene Prediction and KEGG Pathway Enrichment

The target genes for eligible miRNAs were predicted using the miRWalk database (34), and KEGG (Kyoto Encyclopedia of Genes and Genomes) pathway enrichment analysis for the predicted genes was performed using the KOBAS (KEGG Orthology-Based Annotation System) server (35). P values were calculated using the hypergeometric test. The Q value is the P value corrected using the Benjamini-Hochberg method. Pathways with a Q value smaller than 0.01 were considered to be significantly enriched.

### Integrated Analyses of mRNA, lncRNA, circRNA, miRNA, and CNV

mRNA and long non-coding RNA (lncRNA) expression profiles were derived from a previous study (36). And circos plots of CNV (copy number variation) (13) and RNA expression were drawn using the perl package Circos. Up- and down-regulation of different kinds of RNA were determined using the DESeq2 software package as described above.

## Results

### Identification of Circular RNAs in MDA-MB-231 and MCF-7 Breast Cancer Cells and Their Docetaxel-Resistant Sublines

Ribosomal RNA-depleted RNA-sequencing was performed to profile circRNAs in the docetaxel-resistant MDA-RES and MCF7-RES cell lines and their docetaxel-sensitive parental cell lines, MDA-MB-231 and MCF-7, respectively. 3 biological replicates were sequenced for each cell line.

**Figure 1:**
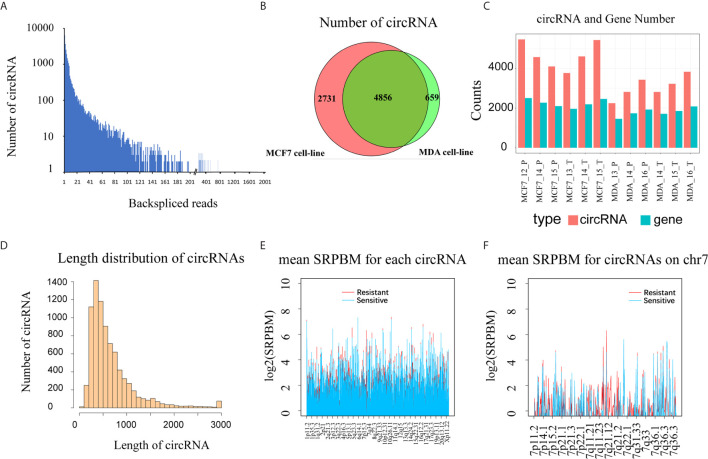
Overview of circular RNAs detected in 12 breast cancer cell samples (three biological replicates for each of the MCF-7, MCF7-RES, MDA-MB-231 and MDA-RES cell lines). **(A)** The back-spliced reads and the number of circRNAs identified in 12 breast cancer cells. **(B)** The intersection of circRNAs detected in 6 MDA cells and 6 MCF cells. **(C)** The number of circRNAs and the number of circRNA genes in each sample. **(D)** The histogram shows the distribution of all detected circRNA lengths. **(E)** The expression level of circRNAs (SRPBM) in different groups throughout the genome. The docetaxel-resistant group is shown in red and docetaxel-sensitive group in blue. **(F)** Expression of circRNAs (SRPBM) in different groups across Chromosome 7. The resistant group is shown in red and the sensitive group in blue.

A total of 1,825,984,984 raw reads were generated by the Illumina HiSeqTM 2000 sequencer for the 12 RNA samples (4 cell lines × 3 biological replicates each), and 1,750,124,272 clean reads (157.5 Gb) were obtained after data filtering, resulting in 146 million clean reads per sample. By mapping the reads to a human reference genome (hg19) with Tophat2 software, and using CIRCexplorer software to identify and annotate circRNAs, we detected 8,246 high-reliability exonic circRNAs from the 12 samples, with at least two unique back-spliced reads in more than one sample (**Figure 1A** and **Supplementary Table 1**). In the MCF7 and MDA cells, 7,588 and 5,515 circRNAs were detected, respectively, and 58.9% of these circRNAs were shared by the two cell lines (**Figure 1B**). In MCF7 cells, the number of circRNAs ranged from 3,770 to 5,472, and the number of genes generating these circRNAs ranged from 1,964 to 2,508, while in MDA cells the number of circRNAs and the genes generating the circRNAs were both lower than in MCF cells. There were no significant differences in the number of circRNAs and circRNA genes between the docetaxel-sensitive and -resistant groups in either of the two cell types (**Figure 1C**). By analyzing the length distribution of the detected circRNAs, we found that 90% were less than 1,200 NT in length and the most abundant circRNAs ranged between 300 to 400 NT (**Figure 1D**). This result is consistent with previous research by Zheng et al. on circRNAs in cancer tissues (21).

To evaluate the expression of circRNAs, we normalized the number of back-spliced reads with the total number of mapped reads and read lengths as described above (21). To get an overview of the distribution and expression level of circRNAs in both the DRBC cell lines and their parental cell lines, we calculated the mean SRPBM for each circRNA in docetaxel-sensitive and -resistant groups across the genome (**Supplementary Table 1**). We found that circRNA expression exhibited significant differences between the docetaxel-sensitive and -resistant groups in some regions of the genome, especially in Chromosome 7 q21.12 to Chromosome 7 q21.2, where the circRNAs were expressed mainly in DRBC cells (**Figures 1E, F**). This result indicates that some circRNAs may be specifically expressed in docetaxel-sensitive or -resistant breast cancer cells.

### Identification of circRNA Signatures and Potential Functional circRNAs in Parental and Docetaxel-Resistant Cell Lines

**Figure 2:**
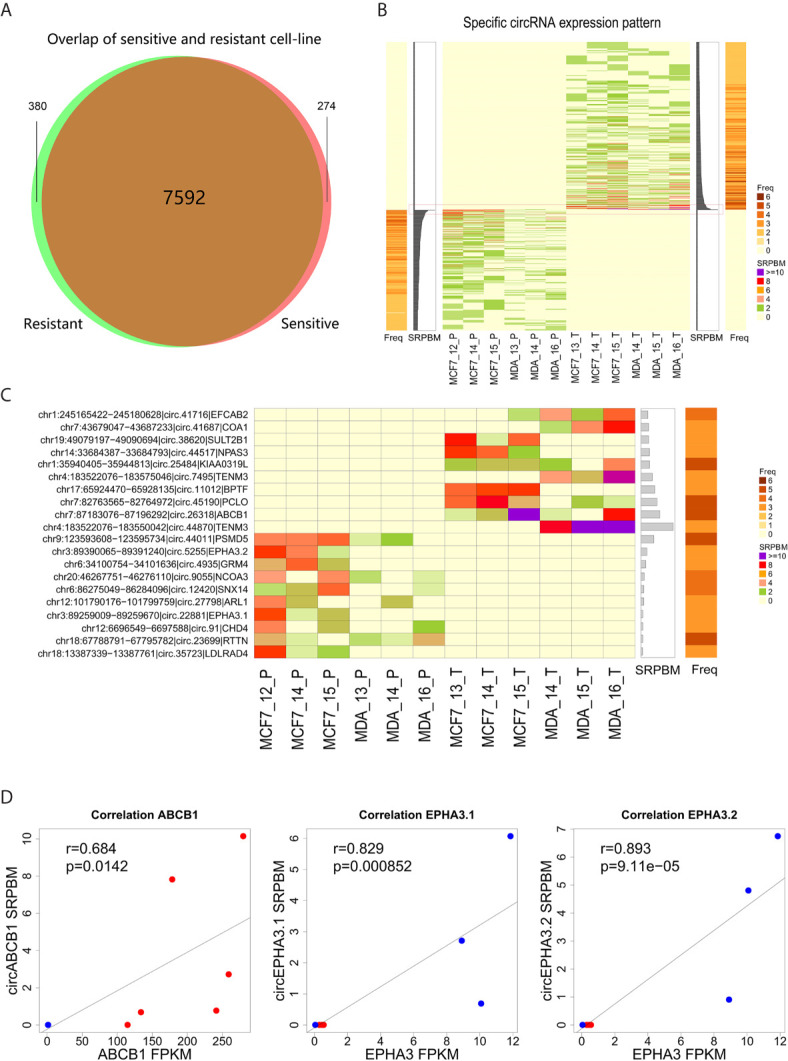
Expression level of circRNA specifically detected in docetaxel-resistant or -sensitive cells. **(A)** The intersection of circRNAs detected in docetaxel-resistant and -sensitive cells after filtering. **(B)** CircRNAs specifically detected in the docetaxel-resistant and docetaxel-sensitive groups. The central heatmap indicates the expression level (SRPBM) of 654 specifically expressed circRNAs in each sample. The bar plot next to the central heatmap indicates the sum of SRPBM in all samples of a circRNA. The bar plots on the left or right indicate the frequency of a circRNA detected in the samples. The red rectangle marks the top 10 circRNAs specifically detected in the docetaxel-resistant or -sensitive groups. **(C)** Detailed information for the top 10 circRNAs specifically detected in the docetaxel-resistant or -sensitive groups. **(D)** Correlation of the three highly abundant circRNAs and their linear isoforms from the same genes.

To identify circRNAs related to docetaxel sensitivity or resistance, we comparatively analyzed the expression of circRNAs in docetaxel-sensitive and -resistant cell lines. We found that 380 circRNAs were specifically expressed in docetaxel-resistant cell lines and 274 circRNAs were specifically expressed in docetaxel-sensitive cell lines (**Figure 2A** and **Supplementary Table 2**). We further estimated the abundance of specifically-expressed circRNAs by calculating their SRPBM values and the expression frequencies in docetaxel-resistant or -sensitive cell lines (**Figure 2B** and **Supplementary Table 2**). The top 10 circRNAs with the highest SRPBM values in docetaxel-resistant and -sensitive cell lines are shown in **Figure 2C**.

Notably, we found that three of the most highly expressed circRNAs were generated by genes closely associated with multi-drug resistance in cancer cells. Circ.26318 is generated by the well-known multi-drug resistance gene *ABCB1* (12, 37). We designated this newly identified circRNA “circABCB1”. The specific detection of circABCB1 in DRBC cells suggests that the expression of this circRNA may be related to the resistant phenotype. Two of the highly abundant circRNAs, circ.22881 and circ.5255, were specifically detected in docetaxel-sensitive parental cells and annotated to the multi-drug resistance gene *EPHA3* (38, 39). We designated these two newly identified circRNAs “circEPHA3.1” and “circEPHA3.2”.

To investigate the relationship between these three circRNAs and their linear isoforms, we performed correlation analysis on the expression data of these three circRNAs and their linear isoforms. We found that the level of circABCB1 was positively correlated with the level of linear ABCB1 transcripts (Pearson correlation test, R=0.684, P=0.0142, **Figure 2D**). Similarly, expression of circEPHA3.1 and circEPHA3.2 were both positively correlated with the expression of the linear EPHA3 transcripts (Pearson correlation test, R=0.829, P=8.5×10^-4^ and R=0.893, P=9.11×10^-5^, **Figure 2D**). These results suggested that there may be a regulatory relationship between these circRNAs and their linear isoforms.

### Genomic Structure Analysis and Validation of circRNAs Generated by *ABCB1* and *EPHA3*

**Figure 3:**
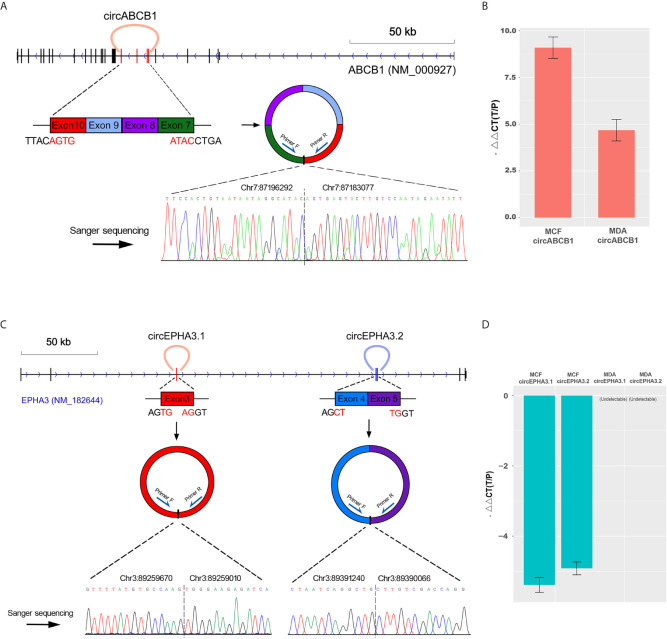
Structure analysis and qPCR validation of circRNAs. **(A)** The upper panel shows the cyclized exons and the structure of circABCB1 in the *ABCB1* gene locus. The bottom panel shows Sanger sequencing of the PCR product of the junction sequence. **(B)** qPCR validation of circABCB1 expression in MCF-7 and MDA cells, with GAPDH used as an endogenous control. The heights of the red columns represent the logarithmically transformed mean fold-changes between the resistant and parental cells detected by qPCR. Data from triplicate measurements are presented as mean ± standard error. **(C)** The upper panel shows the cyclized exons and structures of circEPHA3.1 and circEPHA3.2 in the *EPHA3* gene locus. The bottom panel shows Sanger sequencing of the PCR product of the junction sequence. **(D)** qPCR validation of circEPHA3.1 and circEPHA3.2 expression in MCF-7 and MDA cells, with GAPDH used as an endogenous control. The heights of the blue columns represent the logarithmically transformed mean fold-changes between the resistant and parental cells detected by qPCR. Data from triplicate measurements are presented as mean ± standard error.

We first analyzed the genomic structure of these three circRNAs. Our analyses showed that circABCB1 was formed by exon7, exon8, exon9 and exon10 of the *ABCB1* gene. The 5’ boundary of exon7 joins with the 3’ boundary of exon10 to yield a 661 bp-length lariat structure (**Figure 3A**). The circEPHA3.1 was formed by the junction of the 5’ and the 3’ boundaries of exon3 of the *EPHA3* gene and the length of the circular product was 661 bp. The circEPHA3.2 was formed by the junction of the 5’ boundary of exon4 and the 3’ boundary of exon5 of the *EPHA3* gene and the length of circEPHA3.2 was 492 bp (**Figure 3C**).

To validate the circRNAs generated by the *ABCB1* and *EPHA3* genes, outward-facing primers were designed based on the sequences flanking both sides of the junction points of each circRNA. The three putative circRNAs — circABCB1, circEPHA3.1 and circEPHA3.2 — were validated and quantified with cDNA from the DRBC cells and their parental cells. Furthermore, the amplicons were validated by Sanger-sequencing. The qPCR data confirmed a strong up-regulation of circABCB1 in docetaxel-resistant cells (**Figure 3B**). Since the sequences obtained from Sanger sequencing perfectly mapped onto the flanking sequences on both sides of the junction points of circABCB1 (**Figure 3A**), this demonstrated the existence of this circRNA. Moreover, by performing qPCR, we also found that the expression levels of both circEPHA3.1 and circEPHA3.2 were strongly down-regulated in DRBC cells compared to the docetaxel-sensitive cells (**Figure 3D**). Sanger sequencing on these amplicons also demonstrated the existence of these two circRNAs (**Figure 3C**).

### Prediction of the Interactions of circABCB1, circEPHA3.1 and circEPHA3.2 with miRNA

**Figure 4:**
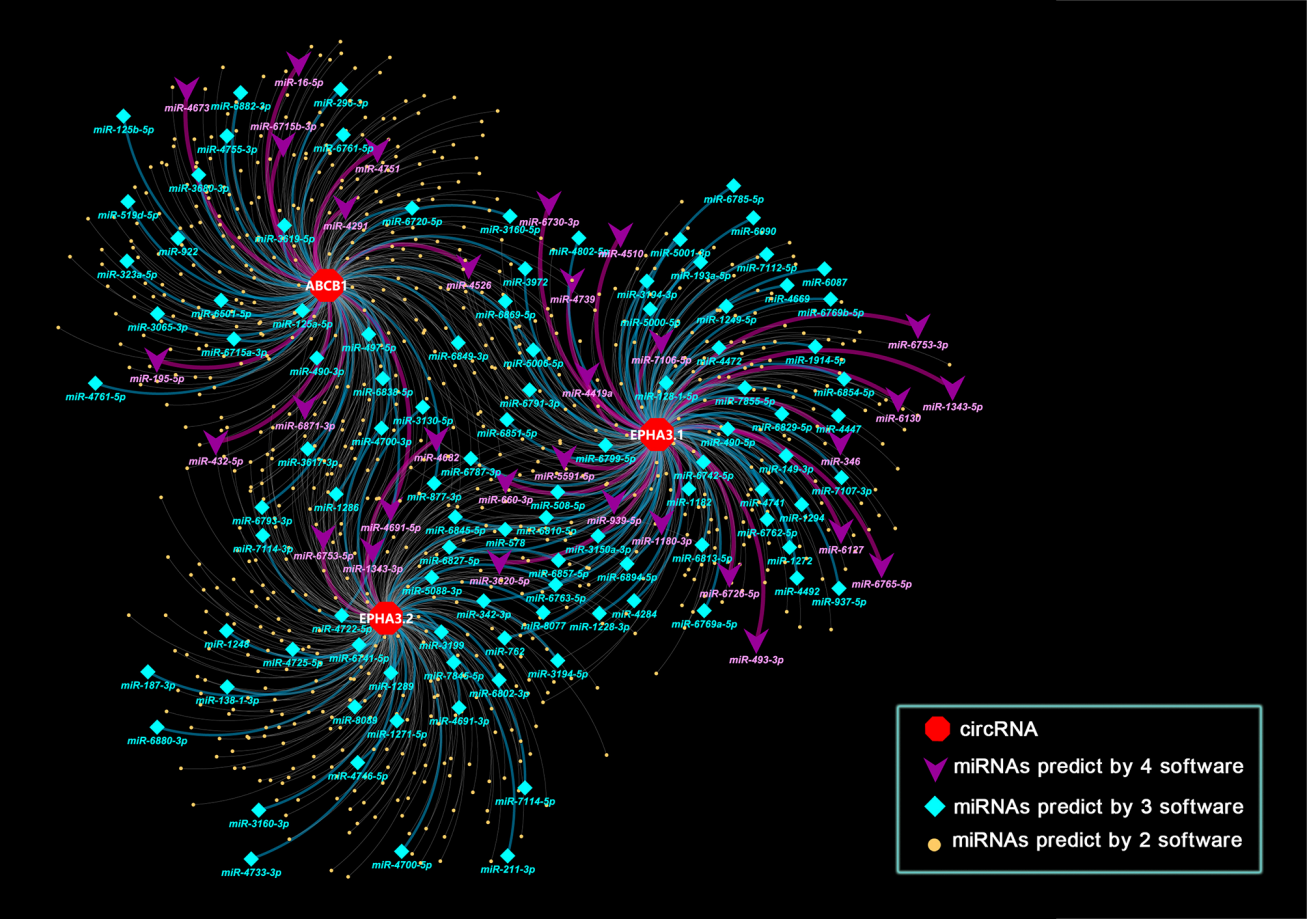
Interaction network of three circRNAs and miRNA. Nodes in the plot represent a circRNA or a miRNA in the interaction network, edges in the plot represent the binding of miRNAs to circRNA. Attributes of the nodes are represented by shape and color. Red hexagons represent circRNAs. Purple triangle nodes indicate microRNAs that target circRNAs as predicted by 4 software tools. Cyan diamond nodes indicate microRNAs that target circRNAs as predicted by 3 software tools. Yellow dots indicate microRNAs that target circRNAs as predicted by 2 software tools. The color of the edges also indicates the number of tools used. Purple indicates that 4 software tools predicted the relationship, while cyan indicates 3 tools and light grey indicates 2 tools.

Since circRNAs bind miRNAs through their miRNA response elements (MREs), we screened the MREs on the sequences of circABCB1, circEPHA3.1 and circEPHA3.2. To improve the accuracy of these predictions, we employed 4 software tools (TargetScan v7.1, Miranda, Pita, and RNAhybrid) to predict potential miRNA target sites on the three circRNAs, and the miRNAs that were predicted to bind to the same circRNA by at least two software tools were chosen as candidate target miRNAs for that circRNA. All the predicted candidate miRNAs are listed in **Supplementary Table 3**. The interaction networks of the three circRNAs and the predicted candidate miRNAs were constructed with Cytoscape. A total of 903 miRNAs were predicted to bind to the three circRNAs, within which 139 miRNAs were predicted by 3 software tools, and 31 miRNAs were predicted by all four of the software tools (**Figure 4** and **Supplementary Table 3**).

### miRNA Sequencing Reveals the Potential Regulation of miRNA Expression by circRNAs in DRBC Cells

To further investigate the interaction of circRNAs and miRNAs, we performed miRNA sequencing using total RNA purified from the MCF-7, MCF7-RES, MDA-MB-231 and MDA-RES cells (three biological replicates were sequenced for each cell line).

**Figure 5:**
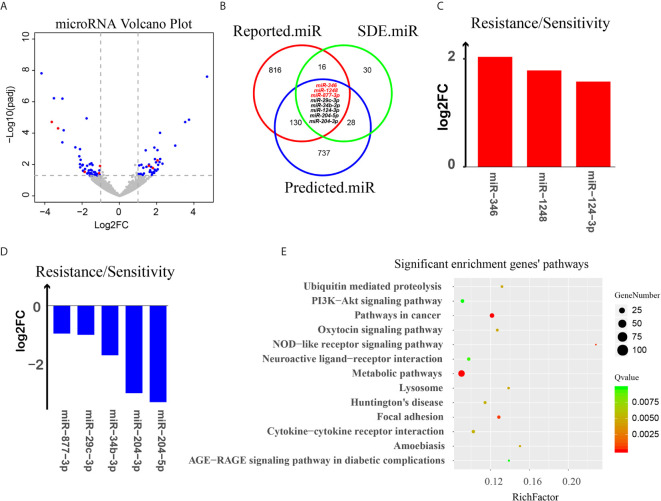
Analysis of miRNA targeting of circRNAs. **(A)** Volcano plot of all miRNAs. The X-axis represents the log_2_(fold-change) of miRNA between docetaxel-resistant cells and -sensitive cells. The Y-axis represents the -log_10_(P value) of the differential expression between the two groups. Blue dots represent miRNAs that were SDE by the two groups. Red dots represent the 8 eligible miRNAs. **(B)** A three-way Venn diagram representing the intersection of 3 miRNA groups: the red cycle represents miRNAs that may relate to chemotherapy reported in PubMed, the green cycle represents SDE miRNAs in our data, and the blue cycle represents miRNAs predicted to target the 3 potentially chemotherapy-resistant circular RNAs, miRNAs predicted by at least three software tools are indicated by red letters. **(C, D)** Log_2_(fold-change) of 8 eligible miRNAs. **(E)** KEGG pathway enrichment for the target genes of the 8 eligible miRNAs.

By performing bioinformatic analysis, we identified a total of 2104 miRNAs from the 12 samples (**Supplementary Table 4**). miRNAs that were differentially expressed between the docetaxel-sensitive and -resistant cell groups were identified using DESeq2 (33). The criteria of |log_2_(fold-change)|>1, P<0.05 was used to select significantly differentially expressed (SDE) miRNAs. We identified 82 SDE miRNAs (44 down-regulated and 38 up-regulated) in DRBC cells by comparing them to docetaxel-sensitive breast cancer cells (**Figure 5A** and **Supplementary Table 5**).

To select the potentially functional SDE miRNAs, we conducted a literature survey of PubMed databases. The miRNAs reported to be associated with chemotherapy resistance were selected and formatted as a reference list and combined with the Pharmaco-miR docetaxel- and paclitaxel-related miRNA list to establish a data source linking miRNAs to chemotherapy resistance and the response to docetaxel and paclitaxel (named Reported.miR, **Supplementary Table 6**).

To investigate the potential relationships of the circRNAs and miRNAs and their contribution to chemotherapy resistance, a three-way Venn diagram was drawn using the SDE miRNAs (SDE.miR), the miRNAs from literature survey (Reported.miR) and the miRNAs predicted to bind to the three circRNAs (Predicted.miR) (**Figure 5B**).

The result showed that there are 24/82 (29%) SDE miRNAs that overlap with the miRNAs in Reported.miR list, indicating that nearly one third of the SDE miRNAs identified in our DRBC cells were associated with response to chemotherapy and may contribute to docetaxel resistance in breast cancer cells (**Supplementary Table 7**). Moreover, there are 36/82 (43.9%) SDE miRNAs that overlap with the miRNAs in the Predicted.miR list, suggested that quite a large portion of SDE miRNAs are predicted by at least two software tools to bind the three circRNAs. More intriguingly, we found that within the 24 SDE miRNAs associated with response to chemotherapy, 8 were also predicted to bind to the three circRNAs by at least two software tools (**Figures 5B–D** and **Table 1**). These results strongly suggest that the three potentially functional circRNAs — circABCB1, circEPHA3.1 and circEPHA3.2 — may contribute to chemotherapy resistance or sensitivity *via* regulation of these miRNAs. We then used the miRWalk database to predict the target genes for the 8 eligible miRNAs, and performed KEGG pathway analysis for the target genes (**Supplementary Table 8**). We found that the target genes for the 8 eligible miRNAs were significantly enriched in 13 signaling pathways (**Figure 5E**). Notably, the PI3K-Akt signaling pathway and AGE-RAGE signaling pathway in diabetic complications, are consistent with pathway enrichment for SDE mRNAs between the docetaxel-sensitive and -resistant cells groups in our previous study (36) (**Supplementary Figures 2A, B**). 

**Table 1:** Differential expression of the 8 miRNAs that were associated with response to chemotherapy and predicted to bind to the three circRNAs by at least two software tools.

miRNA	log_2_FC(Resistant/Sensitive)	P value	circRNAtarget	Publications associated with chemotherapy response
miR-346	2.04	5.51x10^-3^	circEPHA3.1	Du L. et al. (40) Braun FK. et al. (41) Yang et al. (42)
miR-124-3p	1.58	1.22x10^-2^	circEPHA3.2	Liu YX. et al. (43) He C. et al. (44) Khalil S. et al. (45)
miR-204-5p	-3.64	1.95x10^-5^	circEPHA3.2	Bian Z. et al. (46) Yin Y. et al. (47)
miR-1248	1.78	1.69x10^-2^	circEPHA3.2	Xu et al. (48)
miR-204-3p	-3.30	5.01x10^-5^	circEPHA3.2	Chen PH. et al. (49)
miR-34b-3p	-1.87	2.96x10^-2^	circABCB1	Zhou et al. (50) Hermeking et al. (51)
miR-29c-3p	-1.10	3.61x10^-2^	circABCB1	Zhang et al. (52) Ma X. et al. (53)
miR-877-3p	-1.05	1.25x10^-2^	circABCB1	Huang et al. (54) Li et al. (55)

FC, fold-change. The miRNAs differentially expressed between the parental and docetaxel-resistant cell lines were calculated by DESeq2. The P value was calculated based on a negative binomial distribution model.

### Integrated Analyses of mRNA, lncRNA, circRNA and miRNA in Docetaxel Resistant Breast Cancer Cells

**Figure 6:**
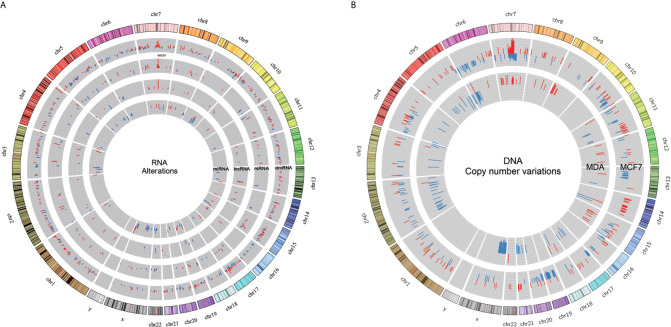
Integrated profile of RNAs and copy number variation. **(A)** Expression profiles of miRNA, lncRNA, circRNA and mRNA in DRBC cell lines. The first (outermost) circle represents the genome location. And the second to the fifth circles show SDE circRNA, mRNA, lncRNA and miRNA. All significantly expressed RNAs were obtained by comparing resistant samples against the corresponding sensitive samples, with red and blue bars for increased and reduced expression, respectively. **(B)** DNA copy number variations in MCF7 and MDA cell lines are depicted by the outer and inner circles, with the blue and red bars representing deletion and amplification, respectively.

mRNA and lncRNA expression data were derived from our previous study that used the same samples (36). We integrated the differential expression data of mRNA, lncRNA, circRNA and miRNA in docetaxel-resistant breast cancer cells and located the RNAs on the whole genome (**Figure 6A**). We also adopted the whole exome sequencing data of (13) and calculated the copy number changes between the docetaxel-resistant and -sensitive cell lines (**Figure 6B**).

By comparing the regions of changes in RNA and DNA copy number, we found that Chromosome 7 q21.12-q21.2 was a common region dominated by DNA amplification and RNA overexpression (**Supplementary Table 9**). Some SDE miRNA and lncRNA loci, and specifically expressed circRNAs, overlap with the CNV regions identified by (13). Intriguingly, we observed DNA copy number gain and overexpression of ABCB1 mRNA and lncRNAs as well as circRNAs specifically expressed in DRBC cells in the *ABCB1* gene locus. It seems reasonable to suggest that overexpression of different RNA molecules in the *ABCB1* gene locus may be caused by amplification of the *ABCB1* gene during exposure to docetaxel (13). Overexpression of ABCB1 mRNA may directly contribute to docetaxel resistance, and overexpression of lncRNAs in the *ABCB1* gene locus was also linked to upregulation of ABCB1 mRNA in our previous study (36).

In this study, we identified specifically expressed circABCB1 in docetaxel-resistant breast cancer cells, and the qPCR experiment confirmed the overexpression of circABCB1 in docetaxel-resistant breast cancer cells in comparison to the sensitive parental cells, which indicated that the expression of circABCB1 in docetaxel-resistant breast cancer cells may also has been caused by the amplification of *ABCB1* gene.

Although we did not find any SDE miRNAs locating within the region of Chromosome 7 q21.12-q21.2. According to the hypothesis of ceRNETs (competing endogenous RNAs networks), some miRNAs may bind to multiple RNA molecules to form competing endogenous RNAs networks and regulate the expression of target genes (56). For example, in our study mir-877-3p was found to bind to both of the circABCB1 and ABCB1 mRNA (**Figure 4**; **Supplementary Table 8**), which indicated that circABCB1 may act as a competing endogenous RNA (ceRNA) to sponge miR-877-3p and led to upregulation of ABCB1 expression and finally contribute to docetaxel resistance in breast cancer cell. Thus, combining observations of changes in DNA and RNA may help to deepen understanding of the mechanisms underlying docetaxel resistance in breast cancer.

## Discussion

Taxanes (including docetaxel and paclitaxel) have been first-line treatments for breast cancer (57). However, inherited or acquired resistance hampers the usefulness of these drugs, and no biomarkers have been identified with sufficient clinical evidence to predict taxane resistance (12).

Although the functions of circRNAs remain largely unknown, recent studies have shown that they can act as microRNA sponges (20) and affect mRNA expression by competing with linear splicing (18). Accumulating evidence also has shown that the circRNA-miRNA-mRNA axis plays important regulatory roles in the development of different types of cancer (22, 58). However, there is still very little data available regarding any roles circRNAs might play in responses to medical treatments. Recently, a study indicated that dysregulation of circRNAs was involved in the development of radiation resistance in esophageal cancer (23) and more recently, three independent studies have linked circRNAs to chemotherapy sensitivity/resistance in acute myeloid leukemia (24), colorectal cancer (59) and breast cancer (25). These findings raise the possibility that circRNAs may also affect the response to docetaxel in breast cancer.

To investigate the potential roles of circRNAs in DRBC cells, we carried out RNA sequencing and analysis of circRNAs in two DRBC cell lines and their parental cell lines. CircRNAs were detected by CIRCexplorer, which is one of the most reliable software tools available for analysis of circRNAs (especially for detection of exonic circRNAs), and these results were used in conjunction with those of four other algorithms (60). In total, we detected 8,246 highly reliable exonic circRNAs from the 12 samples. By comparing the expression of circRNAs in docetaxel-sensitive and -resistant cell lines, 380 circRNAs were found to be specifically expressed in docetaxel-resistant cell lines, and 274 circRNAs were found specifically expressed in docetaxel-sensitive cell lines (**Figures 2A, B**), indicating these circRNAs may be associated with the development of resistance to docetaxel.

Low levels of circRNAs may not be sufficient to affect their target miRNAs (24), and we therefore used SRPBM values to select potentially functional circRNAs by ranking all the specifically expressed circRNAs in both docetaxel-resistant and -sensitive cell lines by their abundance. Notably, among the top 10 most abundant circRNAs in the docetaxel-resistant and -sensitive cell lines, we identified the three most highly expressed circRNAs, which were generated by genes closely associated with docetaxel resistance. Circ.26318 was detected in 5/6 of the DRBC cells and ranked second in the specifically-expressed circRNAs list. Intriguingly, gene annotation revealed that this circRNA was generated by the most studied multi-drug resistance gene, *ABCB1*, which has been reported to be dramatically up-regulated in DRBC cells and recognized as a key mediator of docetaxel resistance (12). We designated the name circABCB1 for this newly identified circular RNA. We found that circABCB1 was formed by the back-spliced junction of exon7 and exon10 of the ABCB1 gene, which results in a 661 bp lariat structure consisting of exon7, exon8, exon9 and exon10. This back-spliced junction was validated by qPCR Sanger sequencing.

It is well documented that circRNAs are not simply the byproducts of mis-splicing (21), and many circRNAs have been shown to play a role in different types of cancer (22, 61). Although the relationships between circular and linear RNA isoforms are largely unknown, it has been shown that circRNAs can regulate genes by competing with linear splicing (18). Interestingly, a recent study has also shown that cir-ITCH increases the expression of ITCH, probably by competing for binding to its associated miRNAs (61). This finding suggests that the functions of circRNAs may be closely associated with those of their linear isoforms. In our study, circABCB1 was specifically detected in DRBC cells by RNA-sequencing, and its levels positively correlated with the linear transcripts of the *ABCB1* gene. These results strongly suggest that circABCB1 may affect the expression of ABCB1 linear transcripts and thus contribute to docetaxel resistance. Therefore, circABCB1 was selected for further investigation as a potential functional circRNA.

Moreover, in the top-10 most abundant circRNAs list for docetaxel-sensitive cell lines, we found that two highly abundant circRNAs, circEPHA3.1 and circEPHA3.2, were specifically expressed in docetaxel-sensitive MCF-7 breast cancer cells, and the expression of these two circRNAs was positively correlated with the linear transcripts of *EPHA3* gene (**Figure 2D**). These two circRNAs were also validated by qPCR Sanger sequencing. *EPHA3* encodes a transmembrane protein that belongs to the ephrin receptor family. This gene has been implicated in various biological processes, including cancer development and progression (62, 63). Recent studies have shown that EPHA3 is down-regulated in chemotherapy-resistant ovarian cancer cells (38) and implicated in regulation of multidrug resistance in small cell lung cancer *via* the PI3K signaling pathway, which has been frequently linked to taxane resistance (39, 64). High expression of circEPHA3.1 and circEPHA3.2 in docetaxel-sensitive breast cancer cells, and their depletion in DRBC cells, indicates that these two circRNAs may be involved in maintaining docetaxel sensitivity in breast cancer cells.

Besides *ABCB1* and *EPHA3*, we found that the annotation genes for the top 10 circRNAs in docetaxel-resistant breast cancer cells are involved in neuronal development, synaptic vesicle trafficking, histone-binding, and maintaining epithelial function *via* the PI3K/AKT signaling pathways. In contrast, those in docetaxel-sensitive breast cancer cells are involved in assembly of the 26S proteasome, G-protein ligand binding, nuclear receptor coactivation, intracellular trafficking, chromatin remodeling, and negative regulation of the TGF-beta signaling pathway (**Supplementary Table 10**). We speculate that some of these genes may be implicated in mediating signal transduction and maintaining the docetaxel-resistant or -sensitive phenotypes in a synergistic way. Although we found none of these genes were directly implicated in docetaxel resistance, some of the genes have been reported to be associated with sensitivity or resistance to chemotherapy or targeted-therapy drugs, such as PCLO, BPTF and CHD4. Thus, the circRNAs generated by these genes may contribute to docetaxel resistance or sensitivity in breast cancer cells. Future functional studies of circRNAs potentially associated with docetaxel resistance in breast cancer cells should take these circRNAs into consideration.

Since acting as an miRNA sponge is currently the most commonly reported function for circRNA (65), and several circRNAs have been shown to bind to miRNAs and thus repress their function (20, 34), we hypothesized that the three candidate circRNAs may act as inhibitors of miRNA and thereby regulate expression of miRNA target genes. Therefore, we performed a bioinformatics analysis to identify miRNA targets for these three circRNAs. As with the database miRWalk (34), this is an effective way to obtain accurate predictions *via* integrating different algorithms. Four prominent target prediction programs, including TargetScan v7.1, Miranda, Pita and RNAhybrid, were employed to predict potential miRNA target sites on the three circRNAs, and finally, 903 overlapping miRNAs predicted by at least two software tools were chosen as candidate targets for the three circRNAs (**Figure 4**).

To further investigate the relationships between the circRNAs and miRNAs, we performed miRNA sequencing in the DRBC cell lines as well as in their parental cell lines, and identified 82 SDE miRNAs. By conducting a literature survey in the PubMed and Pharmaco-miR databases, we found that 29% (24/82) of the SDE miRNAs identified in our DRBC cells were associated with a response to chemotherapy, suggesting that these SDE miRNAs may contribute to docetaxel resistance in breast cancer cells. More intriguingly, eight of these chemotherapy-associated SDE miRNAs were also predicted to bind to the three circRNAs by at least two software tools. These results strongly suggest that the three potential functional circRNAs (circABCB1, circEPHA3.1 and circEPHA3.2) may together regulate the chemotherapy-associated SDE miRNAs and thus affect sensitivity for docetaxel in breast cancer cells.

Among the 8 eligible miRNAs, we found that miR-346 was the only miRNA predicted by all four of the software tools to bind to circEPHA3.1, indicating the robustness of this result. MiR-346 has been shown to act as an oncogenic miRNA in cutaneous squamous cell carcinoma (66) and promote a malignant phenotype in cervical cancer cells (67). More importantly, a recent study showed that the level of miR-346 in breast cancer tissues was higher than in non-cancerous tissues, and overexpression of miR-346 in a breast cancer cell line promoted resistance to docetaxel (42). These reports are consistent with ours in showing that miR-346 is significantly up-regulated in DRBC cells compared with their docetaxel-sensitive parental cell lines (**Figure 5C**), and suggest that the dramatic down-regulation of circEPHA3.1 in DRBC cells may reduce binding and inhibition of miR-346, thus contributing to development of docetaxel resistance.

MiR-1248 was predicted to bind to circEPHA3.2 by three different software tools and was found to be significantly up-regulated in DRBC cells. Although the function of miR-1248 remains largely unknown, it has been implicated in regulating the response to platinum-based chemotherapy (48). It would be interesting to further investigate the relationship between circEPHA3.2 and miR-1248, and to uncover the exact function of miR-1248 in DRBC.

MiR-877-3p, miR-29c-3p, and miR-34b-3p are three miRNAs significantly down-regulated in DRBC cells and predicted to bind to circABCB1 by at least two software tools. By performing a literature survey, we found that miR-877-3p functions as a tumor suppressor and inhibits bladder cancer proliferation (55), consistent with our data showing that over-expression of miR-877 in hepatocellular carcinoma cells increases paclitaxel sensitivity (54). Similarly, low expression of miR-29c was reported to be positively associated with chemotherapy resistance in nasopharyngeal carcinoma patients (52). More interestingly, miR-34b was reported to directly target ABCB1 mRNAs and increase the sensitivity of human osteosarcoma cells to multiple chemotherapeutic agents (50). These findings suggest that circABCB1 probably binds to and down-regulates miRNAs associated with chemotherapy sensitivity in cancer and thus facilitates cancer cell proliferation while contributing to docetaxel resistance in breast cancer. Therefore, the functions of circABCB1 should be explored further.

Finally, to shed some light on the function of the eight eligible miRNAs, we predicted their target genes and performed pathway enrichment analyses for the predicted genes. We identified 13 significantly enriched pathways, and found the PI3K-Akt signaling pathway and AGE-RAGE signaling pathway in diabetic complications to be consistent with the pathway enrichment results for the SDE mRNAs identified in the same cell lines (36) (**Supplementary Figures 2A, B**). In recent years, disruptions of the PI3K-Akt signaling pathway have been frequently identified in cancers, and dysregulation of this pathway has been linked to docetaxel resistance in prostate cancer (68, 69). Although the role of the AGE-RAGE signaling pathway in chemotherapy resistance is largely unknown, it has been reported to activate the PI3K-Akt signaling pathway (70). Taken together, our results indicate that dysregulation of the PI3K-Akt signaling pathway may contribute to the development of docetaxel resistance in breast cancer, and certain circRNA-miRNA-mRNA axes may regulate this pathway.

The current *in vitro* and in silico study is the first to identify circRNAs associated with taxane resistance in breast cancer cells, and we have identified circRNAs generated by well-known multiple drug-resistance genes in DRBC cells and uncovered potential functions of these circRNAs. Also, we obtained a global profile of multi-level RNA alterations in DRBC cells that may lead to a more complete and comprehensive understanding of the mechanisms underlying docetaxel resistance in breast cancer.

These results should be followed up by (1) analyses of some of the identified circRNAs that were not analyzed, (2) experimental investigations of the functions of the circRNAs related to taxane-resistant phenotypes, and (3) analyses of clinical material from breast cancer patients who have relapsed while undergoing taxane treatment.

To sum up, we have performed RNA and miRNA sequencing in two DRBC cell lines and their docetaxel-sensitive parental cell lines. Our analyses revealed different circRNA signatures in the docetaxel-resistant and -sensitive breast cancer cells, and for the first time, uncovered circRNAs generated by multidrug-resistance genes in taxane-resistant cancer cells. CircABCB1 was identified and validated as strongly up-regulated in DRBC cells, whereas circEPHA3.1 and circEPHA3.2 were strongly down-regulated. Furthermore, we investigated potential functions of these circRNAs by bioinformatics analysis, and miRNA analyses were also performed to uncover potential interactions between circRNAs and miRNAs. Our data show that circABCB1, circEPHA3.1 and circEPHA3.2 probably sponge up the eight chemotherapy-associated SDE miRNAs and contribute to docetaxel resistance *via* the PI3K-Akt and AGE-RAGE signaling pathways. Our results should help to deepen understanding of the mechanism of docetaxel resistance in breast cancer and identify novel therapeutic targets or predictive biomarkers for overcoming docetaxel resistance in breast cancer.

## Data Availability Statement

The datasets presented in this study can be found in online repositories (71, 72). The names of the repository/repositories and accession number(s) can be found below: https://db.cngb.org/search/project/CNP0000314/, CNP0000314.

## Author Contributions

JS and PH: conceptualization. CG and CZ: performed sequencing. PH, FL, ZM, FW, and JG: performed data analyses. SW: project funding. PH: wrote the manuscript. JS and LL: revised the manuscript. JS and NB: supervised the project. All authors contributed to the article and approved the submitted version.

## Funding

This work was funded by the National Natural Science Foundation of China (81502412), the Scientific Research Projects of Shenzhen Health System (201501015), and the Guangzhou science and technology program key projects (201604020005).

## Conflict of Interest

Author PH, FL, ZM, CG, FW, CZ, JG and LL were employed by the company BGI-Shenzhen.

The remaining authors declare that the research was conducted in the absence of any commercial or financial relationships that could be construed as a potential conflict of interest.
