# Supplementary material for: A Comprehensive RNA Study to Identify circRNA and miRNA Biomarkers for Docetaxel Resistance in Breast Cancer
Source: Front Oncol. 2021 May 14;11:669270. doi: 10.3389/fonc.2021.669270 (PMC8162208; doi:10.3389/fonc.2021.669270)
Supplement: Supplementary file 1 [file DataSheet_1.docx]

Supplementary Material

# Supplementary Figures and Tables

## Supplementary Figures


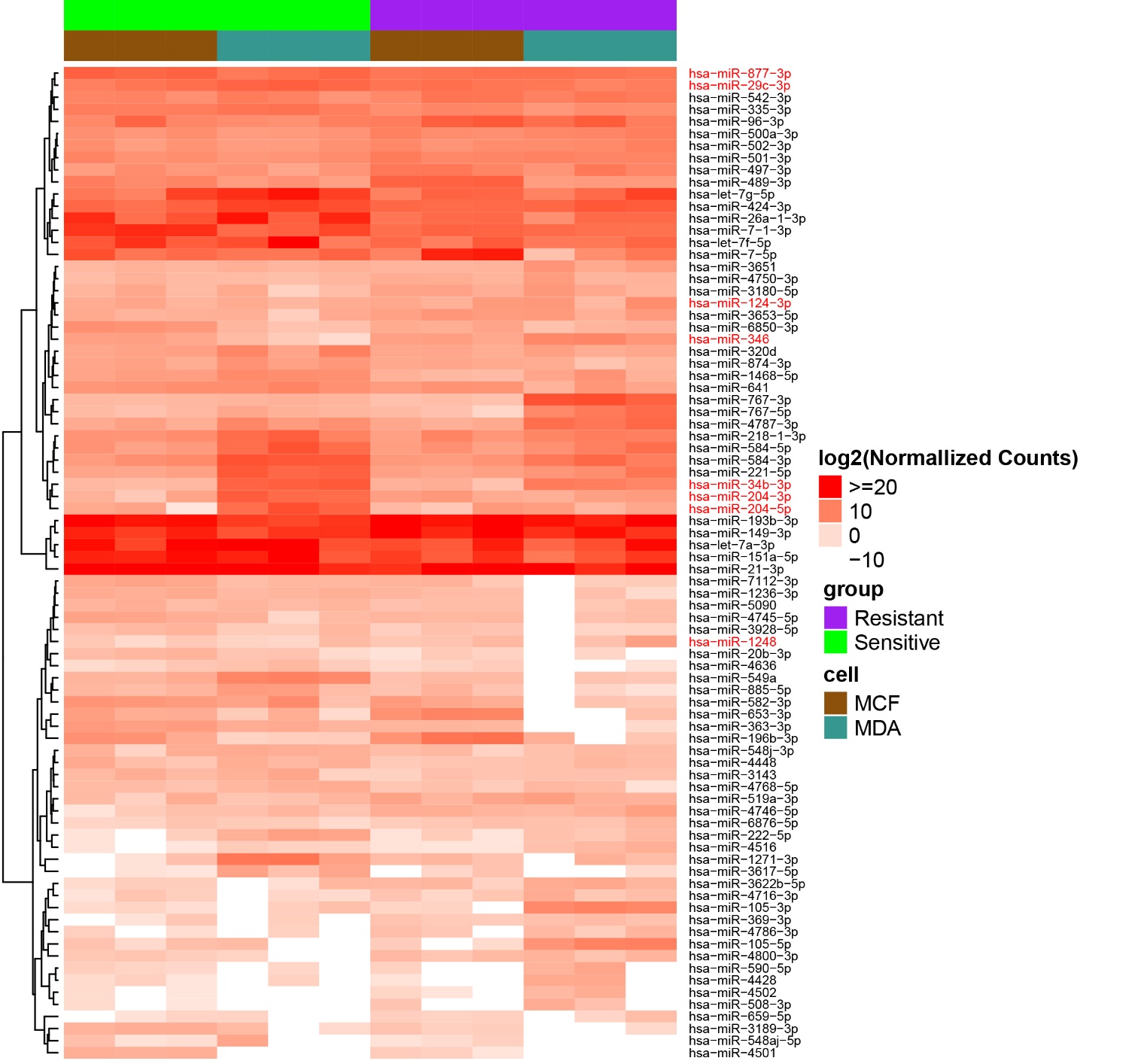


**Supplementary Figure 1.** The heatmap for the 82 SDE miRNAs with highlight of the 8 eligible miRNAs in red.


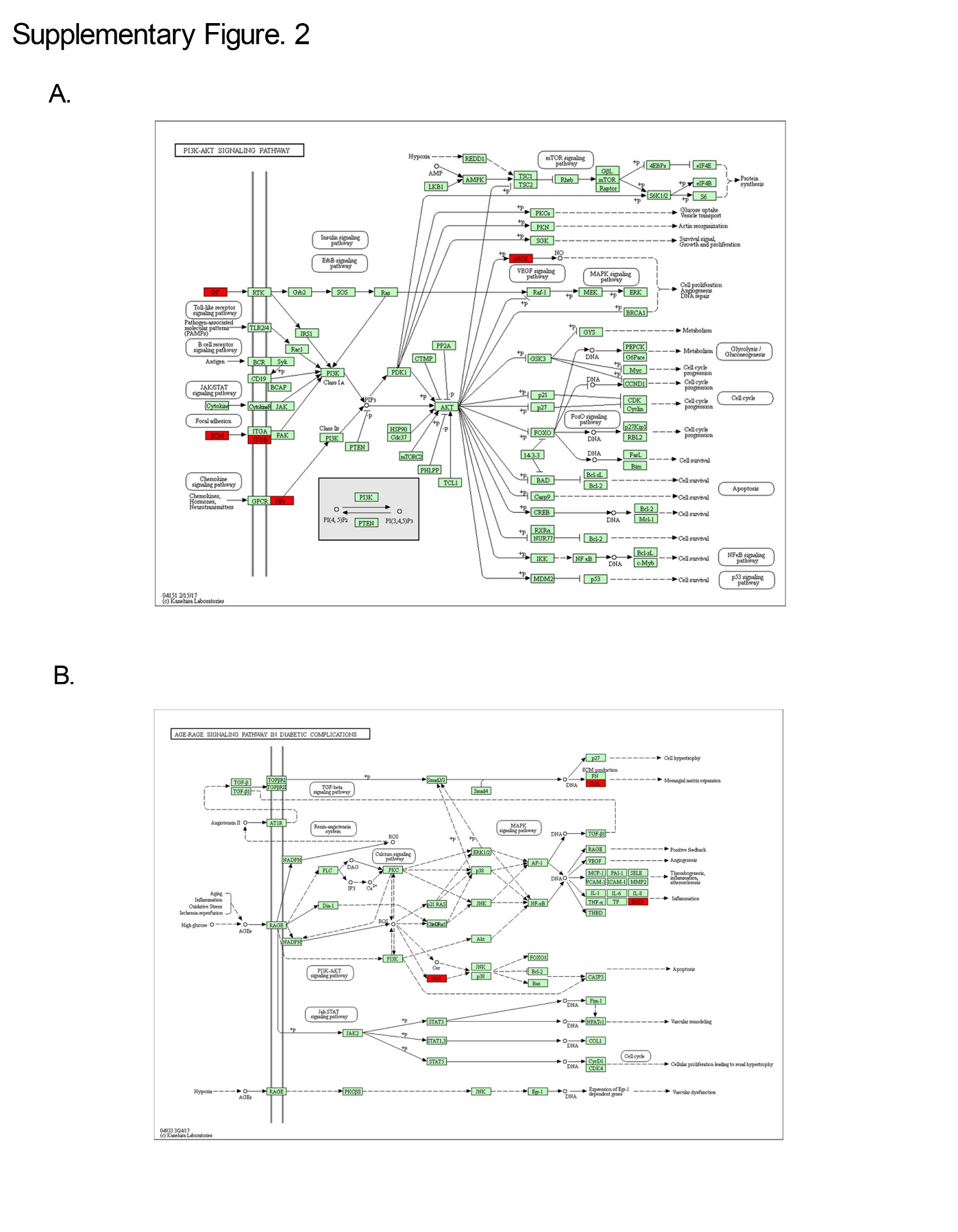


**Supplementary Figure 2.** A. PI3K-AKT signaling pathway for the SDE mRNAs. Rectangles represent all the genes in the pathway, red rectangles: the target genes enrich in the pathway, green rectangles: other genes in the pathway. B. AGE-RAGE signaling pathway in diabetic complications for the SDE mRNAs. Rectangles represent all the genes in the pathway, red rectangles: the target genes enrich in the pathway, green rectangles: other genes in the pathway.

## Supplementary Tables

**Supplementary Table 1.** High reliability exonic circRNAs.

**Supplementary Table 2.** CircRNAs specifically detected in docetaxel resistant or sensitive group.

**Supplementary Table 3.** Prediction of the interactions of circRNAs and miRNAs.

**Supplementary Table 4.** miRNAs detected in 12 samples.

**Supplementary Table 5.** Significantly differential expressed miRNA.

**Supplementary Table 6.** Summary of the literature survey for miRNAs associated with chemotherapy.

**Supplementary Table 7.** SDE miRNAs which were associated with chemotherapy.

**Supplementary Table 8.** Target genes of the 8 miRNA.

**Supplementary Table 9.** The CNVs and SDE or specifically expressed RNAs in Chromosome 7 q21.12-q21.2.

**Supplementary Table 10.** Surveying results of the annotation genes for the top 10 circRNAs in docetaxel-resistant and docetaxel-sensitive breast cancer cells.
